# YAP–VGLL4 antagonism defines the major physiological function of the Hippo signaling effector YAP

**DOI:** 10.1101/gad.350127.122

**Published:** 2022

**Authors:** Jing Cai, Kyungsuk Choi, Hongde Li, Katiuska Daniela Pulgar Prieto, Yonggang Zheng, Duojia Pan

**Affiliations:** Department of Physiology, Howard Hughes Medical Institute, University of Texas Southwestern Medical Center, Dallas, Texas 75390, USA

**Keywords:** Hippo signaling, TEAD, YAP, VGLL4, default repression, tumor suppressor, regeneration

## Abstract

The Hippo–YAP signaling pathway plays a critical role in development, homeostasis, regeneration, and tumorigenesis by converging on YAP, a coactivator for the TEAD family DNA-binding transcription factors, to regulate downstream transcription programs. Given its pivotal role as the nuclear effector of the Hippo pathway, YAP is indispensable in multiple developmental and tissue contexts. Here we report that the essentiality of YAP in liver and lung development can be genetically bypassed by simultaneous inactivation of the TEAD corepressor VGLL4. This striking antagonistic epistasis suggests that the major physiological function of YAP is to antagonize VGLL4. We further show that the YAP–VGLL4 antagonism plays a widespread role in regulating Hippo pathway output beyond normal development, as inactivation of *Vgll4* dramatically enhanced intrahepatic cholangiocarcinoma formation in *Nf2*-deficient livers and ameliorated CCl_4_-induced damage in normal livers. Interestingly, *Vgll4* expression is temporally regulated in development and regeneration and, in certain contexts, provides a better indication of overall Hippo pathway output than YAP phosphorylation. Together, these findings highlight the central importance of VGLL4-mediated transcriptional repression in Hippo pathway regulation and inform potential strategies to modulate Hippo signaling in cancer and regenerative medicine.

## Abstract (precis)

In this study, Cai et al. report that the essentiality of YAP in liver and lung development can be genetically bypassed by simultaneous inactivation of the TEAD corepressor VGLL4, which suggests that the major physiological function of YAP is to antagonize VGLL4. Their findings highlight the central importance of VGLL4-mediated transcriptional repression in Hippo pathway regulation and inform potential strategies to modulate Hippo signaling in cancer and regenerative medicine.

The Hippo signaling pathway acts through a core kinase cascade to control the activity of the transcriptional coactivator YAP (or its paralog, TAZ) in response to various upstream cues (Johnson and Halder 2014; Yu et al. 2015; Davis and Tapon 2019; Zheng and Pan 2019). The core Hippo kinase cascade consists of two tumor suppressor complexes, the MST1/2–SAV1 complex and the LATS1/2–MOB1A/B complex, where the MST1/2–SAV1 complex phosphorylates and activates the LATS1/2–MOB1A/B complex. The activated LATS1/2–MOB1A/B complex then phosphorylates YAP, resulting in YAP cytoplasmic retention and degradation. When Hippo signaling is low, YAP translocates into the nucleus to partner with the DNA-binding transcription factor TEAD1/2/3/4 to activate target gene transcription.

YAP is a well-established oncoprotein whose overexpression and/or aberrant activation have been frequently observed in various tumors due to gene amplification, gene fusion, or mutation of upstream tumor suppressors (Zanconato et al. 2016; Szulzewsky et al. 2021). The supraphysiological YAP activity in tumor cells has been shown to promote the expression of genes involved in initiation, progression, metastasis, and drug resistance, and YAP inhibition is currently being explored for the development of novel anticancer therapeutics. While many of the studies to date have focused on how aberrant YAP activity promotes tumorigenesis, an essential role for physiological YAP activity in the normal development of multiple organs has also been revealed by mouse genetic studies (Davis and Tapon 2019; Zheng and Pan 2019). For example, liver-specific knockout of *Yap* during mouse development results in hypoplastic biliary ducts and hepatomegaly, partially due to increased fibrogenesis (Zhang et al. 2010). Likewise, deletion of *Yap* specifically in the lung epithelium disrupts lung branching morphogenesis, leading to cyst formation (Lin et al. 2017). However, our mechanistic understanding of the genetic circuitry by which physiological YAP activity regulates normal tissue development and the relevance of such genetic circuitry to tumorigenesis or regeneration remains incomplete.

We have previously identified vestigial-like family member 4 (VGLL4) as a transcriptional corepressor that competes with the coactivator YAP for TEAD binding (Koontz et al. 2013). Unlike VGLL1–3 of the family, each of which possesses a single Tondu (TDU) domain, VGLL4 has two TDU domains that bind TEADs in a manner similar to the way that the *Drosophila* ortholog of VGLL4, Tondu domain-containing growth inhibitor (Tgi), interacts with the TEAD ortholog Sd (Mesrouze et al. 2021). Moreover, while VGLL1–3 have been reported to promote cancer cell growth (Deng and Fang 2018), *VGLL4* overexpression completely ameliorated the massive hepatocellular carcinoma formation induced by perinatal *YAP* overexpression in transgenic mice (Koontz et al. 2013). Most interestingly, Tgi is part of a default repression mechanism by which the TEAD ortholog Sd actively represses target gene expression in the absence of Yki, the *Drosophila* ortholog of YAP (Koontz et al. 2013). The importance of this default repression mechanism was supported by genetic epistasis showing that loss of Tgi rescues the loss-of-Yki phenotypes, suggesting that the major function of Yki is to antagonize Tgi-mediated default repression. However, it remains unclear whether a similar default repression mechanism operates in mammals. Meanwhile, increasing evidence has implicated VGLL4 in human cancer, as reduced VGLL4 mRNA and protein levels were observed in various tumors, such as gastric cancer (Jiao et al. 2014), lung cancer (Zhang et al. 2014), esophageal squamous cell carcinoma (Jiang et al. 2015), and colorectal cancer (Jiao et al. 2017). The exact role and mechanism of VGLL4 in tumorigenesis and regeneration remain to be elucidated.

In this study, we generated a conditional *Vgll4* knockout mouse model to investigate the functional relationship between VGLL4 and YAP. We found that loss of *Vgll4* completely rescued the developmental defects of *Yap* mutant livers and lungs, greatly enhanced *Nf2* mutant liver intrahepatic cholangiocarcinoma development, and ameliorated CCl_4_-induced liver injury. Our results thus suggest that the major function of YAP in normal tissue development is to antagonize VGLL4-mediated default repression, and that the YAP–VGLL4 antagonism additionally regulates Hippo signaling output in tumorigenesis and regeneration.

## Results

### Generation of liver-specific *Vgll4* knockout mice

To investigate the role of VGLL4 in development and homeostasis, we generated a conditional knockout allele of *Vgll4* (*Vgll4*^*cKO*^) by inserting two *loxP* sites flanking exon 3 of the *Vgll4* locus through homologous recombination in embryonic stem (ES) cells (Supplemental Fig. S1). A PGK-neomycin cassette used for drug selection was flanked by two *FRT* sites, inserted between exon 3 and the second *loxP* site, and later removed by flippase to generate the *Vgll4*^*flox*^ allele. Subsequent deletion of the targeted exon 3 by Cre recombinase would generate a *Vgll4* knockout allele (*Vgll4*^*KO*^) and inactivate VGLL4 (Supplemental Fig. S1).

**Figure 1.**
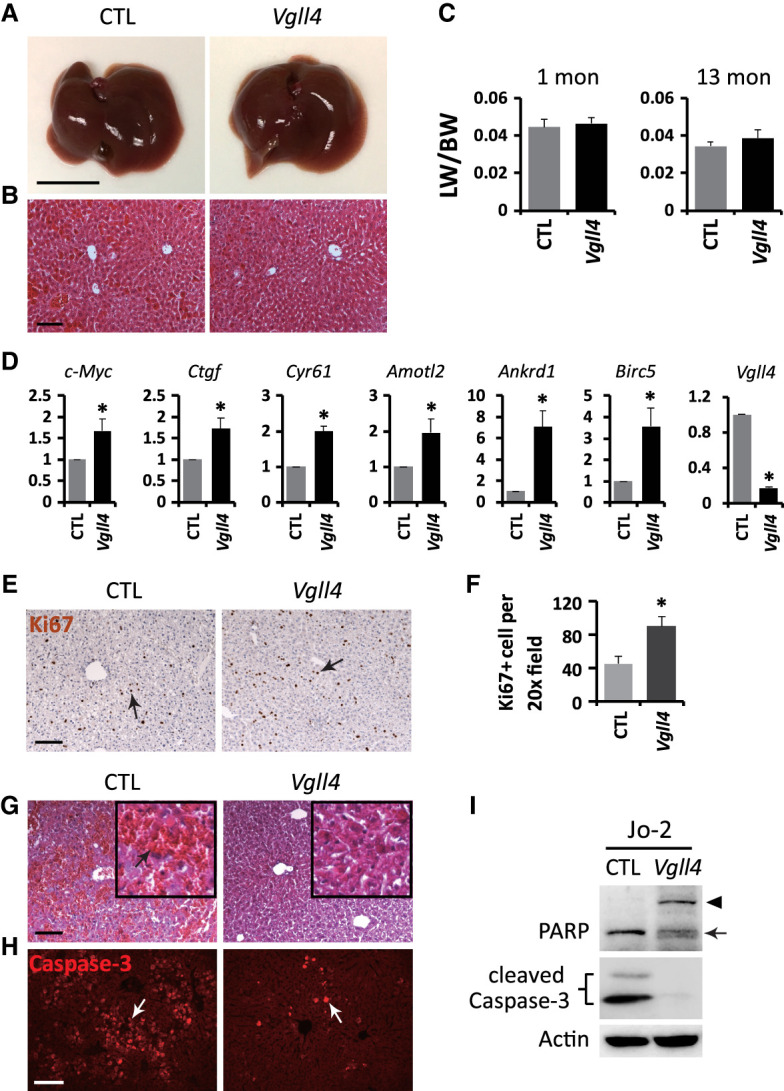
Loss of *Vgll4* increases YAP signaling output in the liver. (*A*) Morphology of 13-mo-old control (*Vgll4*^*flox/flox*^) and *Vgll4* mutant (*Alb-Cre;Vgll4*^*flox/flox*^) livers. Scale bar, 1 cm. (*B*) H&E staining of 13-mo-old control and *Vgll4* mutant livers. Scale bar, 100 μm. (*C*) The ratio of liver weight (LW) over body weight (BW) in control (1 mo, *n* = 9; 13 mo, *n* = 8) and *Vgll4* mutant (1 mo, *n* = 8; 13 mo, *n* = 6) mice. (*D*) Up-regulation of YAP target genes in 1-mo-old *Vgll4* mutant livers. Data are mean ± SD. *n* = 3. (*) *P* < 0.05, *t*-test. (*E*) Ki67 staining (arrows) in 1-mo-old control and *Vgll4* mutant livers. Scale bar, 100 μm. (*F*) Quantification of Ki67-positive cells in *E*. Data are mean ± SD. *n* = 3. (*) *P* < 0.01, *t*-test. (*G*,*H*) H&E and cleaved Caspase-3 (white arrows) staining in livers 3 h after intraperitoneal Jo-2 injection in 3-mo-old control and *Vgll4* mutant mice. The black arrow in *G* indicates liver hemorrhage. Scale bar, 100 µm. (*I*) Western blot showing decreased apoptosis in *Vgll4* mutant livers compared with control livers after Jo-2 treatment. Liver lysates from animals described in *G* and *H* were probed with anti-PARP antibody to detect PARP cleavage or anti-cleaved Caspase-3, both markers of apoptosis. The full-length and cleaved PARPs are indicated by an arrowhead and an arrow, respectively.

*Vgll4*^*flox/flox*^ mice were viable and exhibited no overt abnormalities. However, homozygous mice carrying the *Vgll4*^*KO*^ allele (generated by breeding *Vgll4*^*flox*^ male mice with *Sox2-Cre* female mice) died before embryonic day 11.5 (E11.5). To analyze the function of VGLL4 in the liver, we generated *Alb-Cre;Vgll4*^*flox/flox*^ progenies by breeding *Vgll4*^*flox*^ mice with the *Albumin-Cre* (*Alb-Cre*) driver, in which liver-specific Cre activity was first detected at E13.5, with Cre recombinase expressed in both hepatocyte and biliary cell lineages perinatally and postnatally (Xu et al. 2006). Since VGLL4 protein in the liver was not detectable by Western blot or immunohistochemistry using multiple commercially available antibodies, we confirmed successful deletion of *Vgll4* in *Alb-Cre;Vgll4*^*flox/flox*^ livers at the mRNA level by real-time PCR (Fig. 1D).

### VGLL4 suppresses steady-state YAP signaling output in the liver

*Alb-Cre;Vgll4*^*flox/flox*^ mice were viable and exhibited no overt abnormalities. At 13 mo old, the *Vgll4* mutant livers showed normal morphology, size, and histology compared with the control (Fig. 1A–C). Even by 24 mo old, *Vgll4* mutant livers did not develop tumors or any overt phenotypes. RNA-seq analysis of gene expression profiling revealed only 218 up-regulated genes and 101 down-regulated genes in *Vgll4* mutant livers compared with the control (Supplemental Table S1; Supplemental Fig. S2A). Among the up-regulated genes in *Vgll4* mutant livers, those involved in cell proliferation and immune response are enriched (Supplemental Fig. S2B).

To further investigate whether loss of VGLL4 may result in subtle changes that elude histological analysis, we examined the expression of canonical Hippo pathway target genes. Indeed, loss of VGLL4 mildly increased the expression of canonical YAP target genes such as *c-Myc*, *Ctgf*, *Cyr61*, *Amotl2*, *Ankrd1*, and *Birc5* (Fig. 1D), which was accompanied by increased hepatocyte proliferation (Fig. 1E,F). Several Hippo pathway tumor suppressors, including *Lats2*, *Mst1*, and *Mst2*, were also slightly increased in *Vgll4* mutant livers (Supplemental Fig. S3A), consistent with the well-established negative feedback whereby activation of the nuclear effector YAP leads to up-regulation of upstream tumor suppressors (Chen et al. 2015; Moroishi et al. 2015).

We next examined resistance to Fas-mediated apoptosis, a characteristic hallmark of YAP-activated livers (Dong et al. 2007). While intraperitoneal injection of Fas agonist antibody (Jo-2) induced massive hemorrhage or hepatocyte apoptosis in control livers, the *Vgll4*-deficient livers were greatly protected (Fig. 1G–I), as previously shown in YAP-activated livers. Together, these findings support functional antagonism between VGLL4 and YAP in vivo and are consistent with our previous study implicating VGLL4 and YAP as corepressor and coactivator of the TEAD family transcription factors.

### Loss of VGLL4 bypasses the essential requirement of YAP in normal tissue development

The subtle increase in YAP signaling output in *Vgll4*-deficient mouse livers is reminiscent of *Drosophila*, in which loss of *tgi* does not grossly phenocopy Yki activation, except in a very limited developmental context. Despite the phenotypical “inconsequentiality” upon loss of *tgi*, Tgi inactivation broadly rescued the loss-of-*yki* phenotypes in *Drosophila* (Koontz et al. 2013), suggesting that Tgi mediates the default repressor function of Sd. Whether a similar mechanism operates in mammals is unknown.

**Figure 2.**
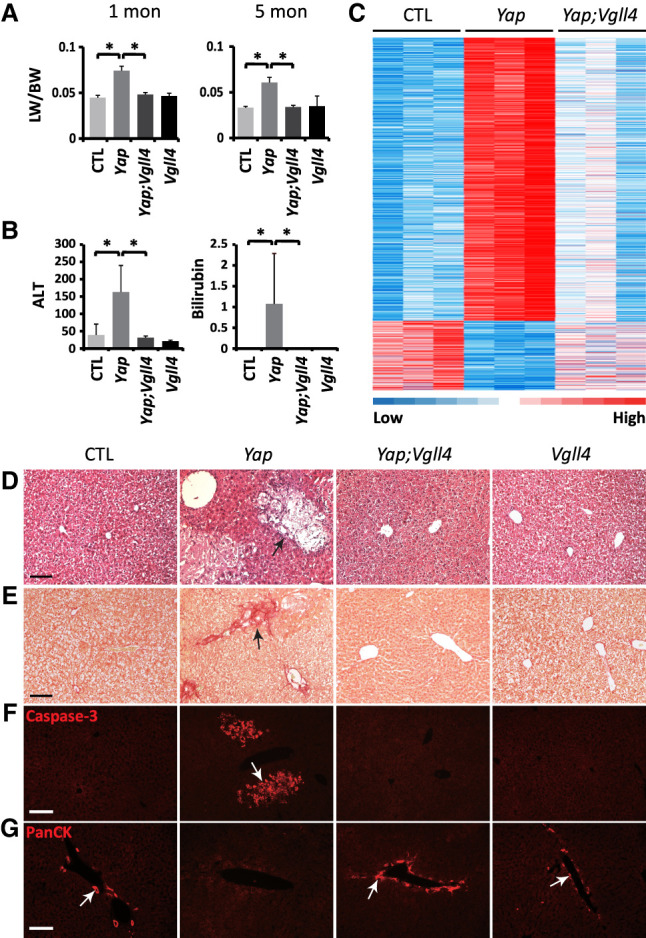
Loss of *Vgll4* completely suppresses *Yap* mutant liver phenotypes. See Supplemental Figure S4 for confirmation of effective deletion of *Yap* and *Vgll4* in *Yap;Vgll4* double-mutant livers. (*A*) The ratio of liver weight (LW) over body weight (BW) in control (*Yap*^*flox/flox*^; 1 mo, *n* = 5; 5 mo, *n* = 4), *Yap* mutant (*Alb-Cre;Yap*^*flox/flox*^; 1 mo, *n* = 5; 5 mo, *n* = 7), *Yap;Vgll4* double-mutant (*Alb-Cre;Yap*^*flox/flox*^*;Vgll4*^*flox/flox*^; 1 mo, *n* = 5; 5 mo, *n* = 6), and *Vgll4* mutant (1 mo, *n* = 8; 5 mo, *n* = 4) mice. Data are mean ± SD. (*) *P* < 0.001, *t*-test. (*B*) Alanine aminotransferase (ALT) and serum bilirubin levels in 2- to 4-mo-old control (*n* = 14), *Yap* mutant (*n* = 16), *Yap;Vgll4* double-mutant (ALT, *n* = 7; bilirubin, *n* = 8), and *Vgll4* mutant (*n* = 6) mice. Data are mean ± SD. (*) *P* < 0.05, *t*-test. (*C*) Heat map analysis of the 2324 up-regulated genes and 558 down-regulated genes (YAP mutant livers vs. control) in control, *Yap* mutant, and *Yap;Vgll4* double-mutant livers. Livers from three independent mice of each genotype were analyzed. (*D*–*G*) H&E, Sirius red, cleaved Caspase-3, and PanCK staining in livers of 1-mo-old control, *Yap* mutant, *Yap;Vgll4* double-mutant, and *Vgll4* mutant mice. The black arrow in *D* and white arrow in *F* indicate hepatocyte apoptosis in *Yap* mutant livers. The black arrow in *E* indicates fibrosis in *Yap* mutant livers. Note the absence of PanCk-positive biliary cells (white arrows in *G*) in *Yap* mutant livers. Scale bar, 100 μm.

The *Vgll4* knockout mice allowed us to formally test the default repression model in mammals. An important prediction of this model is that loss of VGLL4 should bypass the essential requirement of YAP in normal tissue development. We first tested this prediction in the liver, where loss of YAP is known to cause biliary cell loss, which in turn results in defective liver function, hepatocyte death, fibrosis, and compensatory hepatocyte proliferation (Zhang et al. 2010). As reported before (Zhang et al. 2010), liver-specific *Yap* knockout (*Alb-Cre;Yap*^*flox/flox*^) resulted in increased liver size due to compensatory hepatocyte proliferation (Fig. 2A); impaired liver functions, as indicated by increased serum alanine aminotransferase (ALT) and bilirubin levels (Fig. 2B); hepatocyte apoptosis (Fig. 2D,F); fibrosis (Fig. 2E); and loss of the biliary cells (Fig. 2G). While liver-specific *Vgll4* knockout (*Alb-Cre;Vgll4*^*flox/flox*^) exhibited no overt phenotypes on its own, all of these *Yap* knockout phenotypes were completely rescued in *Yap;Vgll4* double-mutant livers (*Alb-Cre;Yap*^*flox/flox*^*;Vgll4*^*flox/flox*^) (Fig. 2A–F; Supplemental Fig. S4). RNA-seq analysis of gene expression profiling in control and mutant livers further confirmed the rescue of *Yap* mutant phenotypes by inactivation of Vgll4. A total of 2324 genes were up-regulated and 558 genes were down-regulated in *Yap* mutant livers compared with the control (Supplemental Table S2). The aberrant expression of these genes was largely rescued to normal levels in *Yap;Vgll4* double-mutant livers (Fig. 2C; Supplemental Table S2). Thus, *Vgll4* is genetically epistatic to *Yap* in the liver.

**Figure 3.**
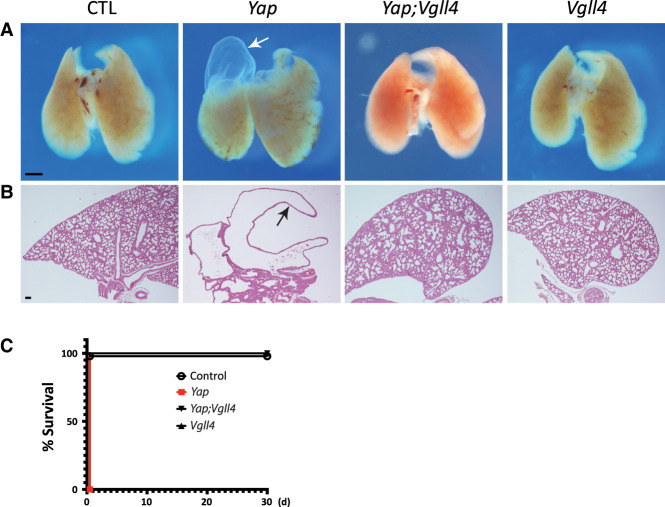
Loss of *Vgll4* rescues *Yap* mutant lung phenotypes. (*A*) Morphology of embryonic day 17.5 (E17.5) control (*Yap*^*flox/flox*^), *Yap* mutant (*Nkx2.1-Cre;Yap*^*flox/flox*^), *Yap;Vgll4* double-mutant (*Nkx2.1;Yap*^*flox/flox*^*;Vgll4*^*flox/flox*^), and *Vgll4* mutant (*Nkx2.1;Vgll4*^*flox/flox*^) lungs. The white arrow indicates lung cyst formation. Scale bar, 100 μm. (*B*) H&E staining in E17.5 control, *Yap* mutant, *Yap;Vgll4* double-mutant, and *Vgll4* mutant lungs. Note the cyst formation in *Yap* mutant lungs (arrow). Scale bar, 100 μm. (*C*) Rescue of *Yap* mutant mice lethality by inactivation of Vgll4 (control, *n* = 18; *Yap* mutant, *n* = 5; *Yap;Vgll4* double mutant, *n* = 17; *Vgll4* mutant, *n* = 13).

To examine whether the genetic epistasis between VGLL4 and YAP observed above is a general feature of the VGLL4–YAP circuitry, we extended our double-mutant analysis to the lung, another organ where YAP is known to play an essential role in development (Lin et al. 2017). For this purpose, we took advantage of a published lung-specific *Yap* knockout model (*Nkx2.1-Cre;Yap*^*flox/flox*^) (Lin et al. 2017; Volckaert et al. 2019), in which the *Nkx2.1-Cre* driver expresses Cre recombinase in the developing lung epithelium as early as E14.5 (Xu et al. 2008). As reported before (Lin et al. 2017; Volckaert et al. 2019), deletion of YAP by the *Nkx2.1-Cre* driver caused cyst formation and perinatal lethality (Fig. 3A–C). While lung-specific *Vgll4* knockout mice (*Nkx2.1-Cre;Vgll4*^*flox/flox*^) were phenotypically normal compared with the control, both cystogenesis and perinatal lethality resulting from lung-specific *Yap* knockout were completely rescued in the *Yap;Vgll4* double mutant (*Nkx2.1-Cre;Yap*^*flox/flox*^*;Vgll4*^*flox/flox*^) (Fig. 3A–C). Together, these results implicate YAP–VGLL4 antagonism as a general mechanism underlying mammalian Hippo signaling in multiple tissue contexts.

### Loss of VGLL4 dramatically enhances tumorigenesis in *Nf2* mutant livers

**Figure 4.**
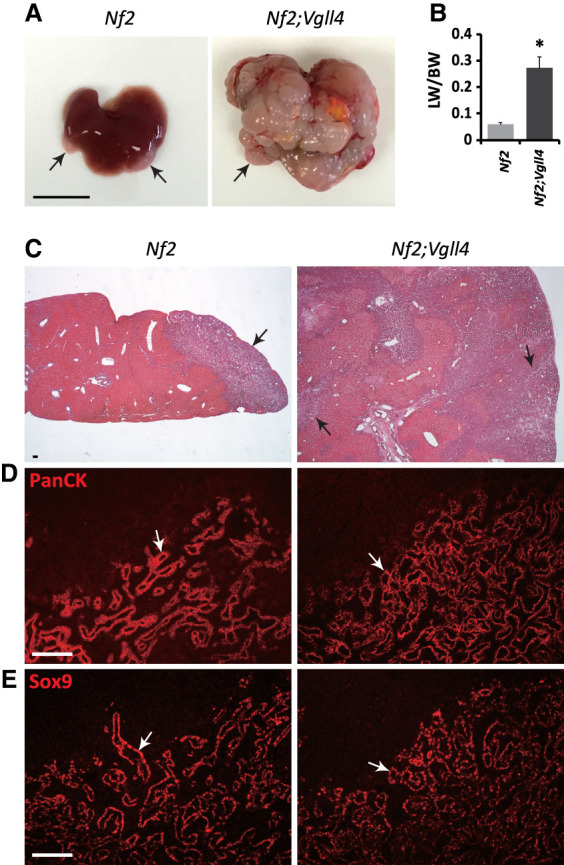
Loss of *Vgll4* dramatically enhances intrahepatic cholangiocarcinoma (ICC) development in *Nf2* mutant livers. (*A*) Morphology of 1-mo-old *Nf2* mutant (*Alb-Cre;Nf2*^*flox/flox*^) and *Nf2;Vgll4* double-mutant (*Alb-Cre;Nf2*^*flox/flox*^*;Vgll4*^*flox/flox*^) livers. Black arrows indicate tumors. Scale bar, 1 cm. (*B*) The ratio of liver weight (LW) over body weight (BW) in 1-mo-old *Nf2* mutant (*n* = 8) and *Nf2;Vgll4* double-mutant (*n* = 9) livers. Data are mean ± SD. (*) *P* < 0.01, *t*-test. (*C*–*E*) H&E, PanCK, and Sox9 staining in 1-mo-old *Nf2* mutant and *Nf2*;*Vgll4* double-mutant livers. Black arrows in *C* and white arrows in *D* and *E* indicate ICCs. Scale bar, 100 μm.

The functional antagonism between VGLL4 and YAP suggests that under normal physiological conditions, the steady-state YAP signaling output is restrained by two distinct mechanisms, one of which is through Hippo signaling-mediated YAP phosphorylation and the other is through corepressor VGLL4 competing with YAP to directly modulate TEAD-mediated transcription (i.e., YAP phosphorylation-independent). A prediction of this model is that inactivation of both VGLL4 and canonical Hippo signaling may have synergistic effects. We tested this prediction by generating *Nf2;Vgll4* double-mutant livers (*Alb-Cre;Nf2*^*flox/flox*^*;Vgll4*^*flox/flox*^). As reported before (Zhang et al. 2010), 1-mo-old *Nf2* single-mutant livers (*Alb-Cre;Nf2*^*flox/flox*^) developed focal intrahepatic cholangiocarcinoma (ICC) at the surface of the liver (Fig. 4A). These tumors were positive for the biliary cell markers PanCK and Sox9 (Fig. 4C–E). In comparison, age-matched *Nf2;Vgll4* double-mutant livers were much larger than *Nf2* single-mutant livers (Fig. 4A,B) and developed massive ICCs that invaded deeply throughout the liver parenchyma (Fig. 4A,C–E). Consistent with the enhanced tumorigenesis, mice with *Nf2;Vgll4* double-mutant livers never survived beyond 6 wk of age, whereas mice with *Nf2* single-mutant livers all survived to at least 1 yr of age. Taken together, these results suggest that the canonical Hippo pathway (YAP phosphorylation-dependent) and the corepressor VGLL4 (YAP phosphorylation-independent) both contribute to restraining the YAP signaling output in vivo. Furthermore, VGLL4 restrains YAP signaling output even when YAP is aberrantly activated as in *Nf2*-deficient livers.

### VGLL4 inactivation improves regeneration after CCl_4_-induced liver injury

The Hippo–YAP pathway is an essential regulator of tissue regeneration (Moya and Halder 2019). In the liver, YAP is transiently activated after bile duct ligation-induced, partial hepatectomy-induced, or carbon tetrachloride (CCl_4_)-induced injury, and accordingly, YAP inactivation compromises liver regeneration following the respective injury (Bai et al. 2012; Lu et al. 2018; Verboven et al. 2021). Conversely, genetic or pharmacological inhibition of Mst1/2 improves regeneration after acute or chronic liver damage (Fan et al. 2016; Loforese et al. 2017).

**Figure 5.**
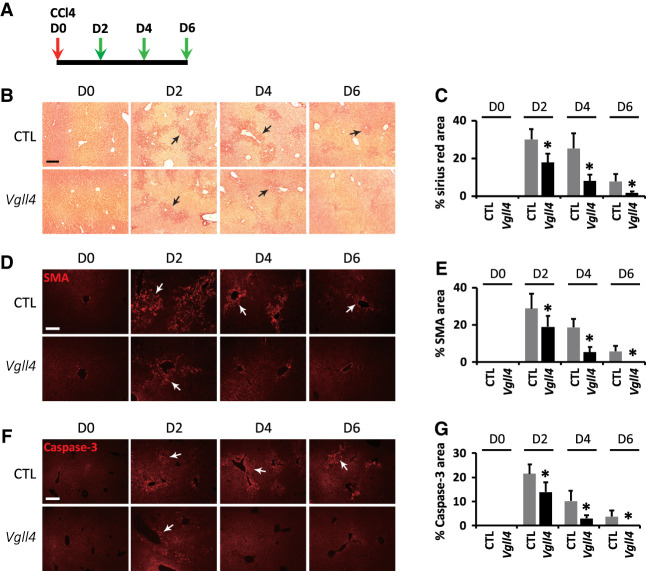
Loss of *Vgll4* improves liver regeneration after CCl_4_ treatment. (*A*) Experimental scheme for CCl_4_-induced liver regeneration. Three-month-old control (*Vgll4*^*flox/flox*^) and *Vgll4* mutant (*Alb-Cre;Vgll4*^*flox/flox*^) mice were injected with one dose of CCl_4_. Livers were harvested 0, 2, 4, and 6 d after CCl_4_ injection and analyzed in *B*–*G*. (*B*–*G*) Sirius red, α-smooth muscle actin (α-SMA), and cleaved Caspase-3 staining in control and *Vgll4* mutant regenerating livers. Damaged areas (arrows) are indicated by Sirius red (*B*), α-SMA (*D*), and cleaved Caspase-3 (*F*) staining and are quantified (*C*,*E*,*G*). Data are mean ± SD. *n* = 3. (*) *P* < 0.01, *t*-test.

Given the functional antagonism between VGLL4 and YAP, we wondered whether loss of VGLL4 also improves liver regeneration. We tested this hypothesis using the CCl_4_-induced acute liver injury model (Liedtke et al. 2013). Three-month-old control or age-matched littermates with liver-specific *Vgll4* knockout were subjected to a single intraperitoneal injection of CCl_4_, and the livers were harvested 2, 4, and 6 d after injection (Fig. 5A). In control mice, histological markers of liver damage, such as the fibrosis markers Sirius red and α-SMA or the apoptosis marker cleaved Caspase-3, were strongly induced 2 d after CCl_4_ injection. These liver damage markers gradually decreased at 4 and 6 d after CCl_4_ injection but were still detectable at 6 d after injection (Fig. 5B–G). In comparison, age-matched littermates with liver-specific *Vgll4* knockout subjected to the same treatment regime exhibited much lower levels of liver fibrosis and apoptosis at all time points (Fig. 5B–G). Thus, VGLL4 inactivation improves regeneration after CCl_4_-induced liver injury, consistent with VGLL4 restraining YAP signaling output in mouse livers.

### Temporal regulation of *Vgll4* expression in development and regeneration

Our studies so far demonstrate that the signaling output of YAP in vivo is regulated by two distinct mechanisms: via Hippo kinase cascade-mediated YAP phosphorylation or corepressor VGLL4, respectively. To further interrogate how the dual mechanisms coordinately control YAP signaling output, we took advantage of the CCl_4_-induced liver regeneration model to compare the temporal patterns of YAP phosphorylation, Vgll4 expression, and YAP target gene expression (as a readout of YAP signaling output).

**Figure 6.**
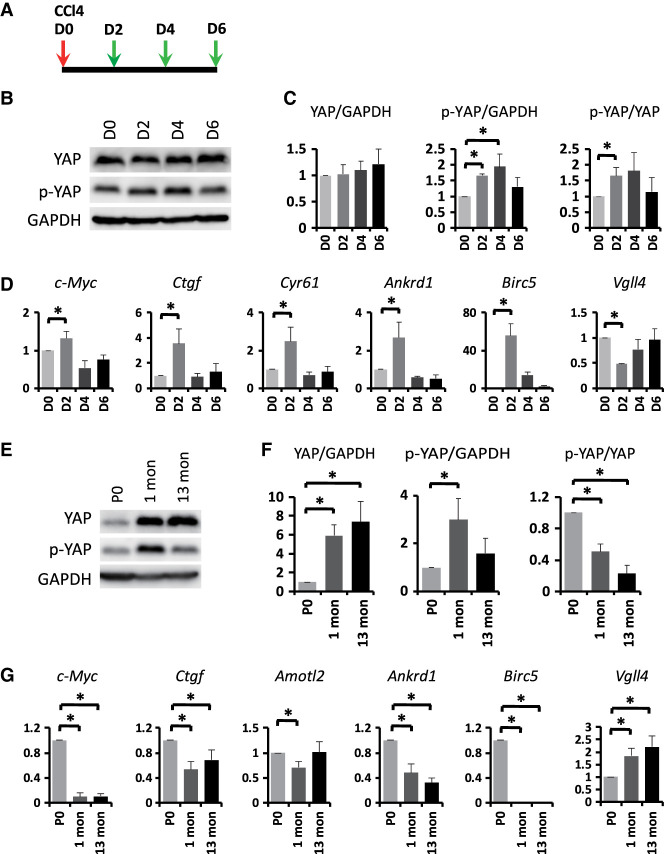
Temporal regulation of *Vgll4* expression in development and regeneration. (*A*) Experimental scheme for CCl_4_-induced liver regeneration. Three-month-old control (*Vgll4*^*flox/flox*^) mice were injected with one dose of CCl_4_. Livers were harvested 0, 2, 4, and 6 d after CCl_4_ injection and analyzed in *B*–*D*. (*B*) Western blot showing unaltered YAP protein levels and increased YAP phosphorylation in regenerating livers. (*C*) Quantification of the ratios of YAP over GAPDH, p-YAP over GAPDH, and p-YAPS112 over YAP. Data are mean ± SD. *n* = 3. (*) *P* < 0.05, *t*-test. (*D*) Up-regulation of YAP target genes and decreased *Vgll4* expression in regenerating livers. Data are mean ± SD. *n* = 3. (*) *P* < 0.05, *t*-test. (*E*) Western blot showing increased YAP protein levels and decreased YAP phosphorylation in adult livers. (*F*) Quantification of the ratios of YAP over GAPDH, p-YAP over GAPDH, and p-YAPS112 over YAP. Data are mean ± SD. *n* = 3. (*) *P* < 0.05, *t*-test. (*G*) Down-regulation of YAP target genes and increased *Vgll4* expression in adult livers. Data are mean ± SD. *n* = 3. (*) *P* < 0.05, *t*-test.

We noted that total YAP protein levels were unaltered 2, 4, and 6 d after CCl_4_ treatment (Fig. 6B,C). However, a transient increase in phospho-YAP levels was observed 2 and 4 d after CCl_4_ injection (Fig. 6B,C), suggesting a transient increase in Hippo signaling. Despite the transient increase of YAP phosphorylation at these time points, which would typically indicate decreased YAP activity, the expression of canonical YAP target genes such as *c-Myc*, *Ctgf*, *Cyr61*, *Ankrd1*, and *Birc5* was paradoxically increased 2 d after CCl_4_ injection. This transient increase in YAP signaling output was accompanied by a transient decrease in *Vgll4* expression (Fig. 6D). YAP phosphorylation, *Vgll4* expression, and YAP target gene expression were all restored to preinjury levels 6 d after CCl_4_ injection (Fig. 6B–D). Thus, in CCl_4_-treated livers, the temporal change of YAP signaling output (increased YAP target gene expression at 2 d after treatment) correlates with that of *Vgll4* expression (decreased Vgll4 expression at 2 d after treatment), not YAP phosphorylation (increased at 2 d after treatment).

We next extended similar analysis to another physiological context: the transition from neonatal to adult livers. Adult livers show increased YAP protein levels with relatively decreased YAP phosphorylation compared with neonatal livers (Fig. 6E,F). While these results would typically indicate an increase in YAP activity in adult livers compared with newborns, the expression of canonical YAP target genes such as *c-Myc*, *Ctgf*, *Amotl2*, *Ankrd1*, and *Birc5* was paradoxically decreased in adult livers (Fig. 6G). Interestingly, this decrease in YAP target gene expression was accompanied by an increase in *Vgll4* expression in adult livers (Fig. 6G). Thus, in the developmental transition from neonatal to adult livers, the temporal change of YAP signaling output (decreased YAP target gene expression in adults) correlates with that of *Vgll4* expression (increased *Vgll4* expression in adults), not YAP phosphorylation (decreased in adult livers).

Taken together, these results suggest that, under certain physiological conditions, *Vgll4* expression may provide a better indication of Hippo–YAP signaling output than YAP phosphorylation.

## Discussion

While VGLL4 has been implicated as a TEAD corepressor based on overexpression studies (Koontz et al. 2013), the endogenous role of VGLL4 in mediating Hippo signaling output remained unclear. In this study, we report the striking finding that loss of VGLL4 rescued the loss-of-YAP defects in liver and lung development, suggesting that the major physiological function of YAP is to antagonize VGLL4-mediated default repression. Given the redundant and distinct functions of YAP's paralog, TAZ, in different tissues, it will be interesting to examine the relevance of TAZ–Vgll4 antagonism in future studies. We further show that both YAP phosphorylation-dependent (via canonical Hippo kinase cascade) and YAP phosphorylation-independent (via VGLL4) mechanisms contribute to restricting Hippo pathway output in vivo. The physiological importance of this dual mechanism is supported by our findings that loss of *Vgll4* dramatically enhanced intrahepatic cholangiocarcinoma formation in *Nf2*-deficient livers. We note, however, that loss of VGLL4 has a relatively milder phenotype than loss of the canonical Hippo kinase cascade, as, unlike the latter, loss of VGLL4 does not cause overt liver abnormality even though it causes modest changes in YAP target gene expression. This is reminiscent of *Drosophila*, in which loss of the VGLL4 counterpart Tgi does not cause visible phenotypes in most developmental contexts. Thus, under normal physiological conditions, kinase cascade-mediated YAP phosphorylation plays a predominant role in dictating the signaling output of the Hippo pathway.

While both VGLL4 and Hippo-mediated phosphorylation contribute to restricting Hippo pathway output in vivo, these two mechanisms are not independent of each other. The increased expression of Hippo pathway tumor suppressors in *Vgll4* mutant livers (Supplemental Fig. 3A) suggests that these mechanisms are functionally coupled and may explain the relative subtle mutant phenotype in *Vgll4* mutant livers. Conversely, we also found that *Vgll4* expression was increased in *Nf2* mutant livers (Supplemental Fig. 3B). The reciprocal, compensatory increase of VGLL4 or Hippo pathway tumor suppressors when the other mechanism is disabled further supports the central importance of the dual mechanisms in Hippo pathway regulation.

Current research frequently uses YAP protein level, subcellular localization, or Hippo-mediated phosphorylation as a readout of Hippo–YAP signaling in tissue homeostasis and pathogenesis. Interestingly, we found that YAP protein level and phosphorylation are uncoupled from YAP target gene expression under certain physiological conditions. For instance, in both CCl_4_-induced liver regeneration and maturation from neonatal to adult livers, the temporal pattern of Hippo–YAP signaling output, as indicated by YAP target gene expression, correlates better with that of *Vgll4* expression than with that of YAP phosphorylation. Thus, spatiotemporal regulation of *Vgll4* expression may provide a previously underappreciated route to modulate Hippo signaling output. While future studies are required to further substantiate this hypothesis, our findings suggest that YAP protein level, localization, or phosphorylation alone does not always serve as a reliable indicator of Hippo–YAP signaling output in vivo.

Another implication of our current study is that VGLL4 may represent a novel target to modulate YAP signaling output in cancer treatment or regenerative medicine. Indeed, VGLL4 has been reported to be down-regulated in multiple cancer types, and enhancing VGLL4 function in these tumors can suppress tumorigenesis (Jiao et al. 2014, 2017; Zhang et al. 2014; Jiang et al. 2015; Kim et al. 2020). Conversely, while ongoing efforts in exploiting the Hippo pathway in regenerative medicine have focused on inhibition of the Hippo kinase cascade to activate YAP (Fan et al. 2016; Loforese et al. 2017), we suggest that VGLL4 inhibition may provide an independent route to enhance YAP signaling output. Our findings that VGLL4 inactivation improves liver regeneration after CCl_4_-induced liver injury provide a proof of concept for further exploring this idea.

## Materials and methods

### Mice

To generate *Vgll4* conditional knockout mice, the targeting vector was constructed using the recombineering technique as described previously (Liu et al. 2003). Briefly, a 9625-bp genomic DNA fragment containing exons 3–5 of the *Vgll4* gene was retrieved from BAC clone RP24-307A13. A *loxP* site was inserted into the intron 2579 bp upstream of exon 3. An *frt-neo-frt-loxP* cassette was inserted into the intron 552 bp downstream from exon 3. The targeting vector was electroporated into a homemade embryonic stem (ES) cell line that was derived from the F1 hybrid blastocyst of 129S6 × C57BL/6J mice. The G418-resistant ES clones were screened by nested PCR, and the positive ES clones were expanded. Chimeric mice were generated by aggregating ES cells with eight-cell embryos of the CD-1 strain. The neo cassette was removed by breeding germline chimeras with *ROSA26-FLP1* homozygous females. The F1 pups were genotyped by PCR using primers flanking the 5′ *loxP* site. The primers used for the *loxp* site were *Vgll4 loxp gtF* (5′-AGCTGAGCTTGGAACACCTT-3′) and *Vgll4 loxp gtR* (5′-TGTGTAAGATGGCAGCCAGT-3′). The PCR products were 238 bp for the wild-type allele and 332 bp for the floxed allele.

*ROSA26-FLP1* (Farley et al. 2000), *Sox2-Cre* (Hayashi et al. 2002), *Albumin-Cre* (*Alb-Cre*) (Yakar et al. 1999), and *Nkx2.1-Cre* (Xu et al. 2008) mice were from the Jackson Laboratory. *Yap*^*flox*^ (Zhang et al. 2010) and *Nf2*^*flox*^ (Giovannini et al. 2000) mice were described previously. These mice were maintained on a mixed genetic background of C57BL/6J and 129/Sv. Mice with *Vgll4*, *Nf2*, or *Yap* specifically deleted in the liver were generated by breeding *Vgll4*^*flox*^, *Nf2*^*flox*^, or *Yap*^*flox*^ mice with *Alb-Cre* mice. Mice with *Vgll4* or *Yap* specifically deleted in the lung were generated by breeding *Vgll4*^*flox*^ or *Yap*^*flox*^ mice with *Nkx2.1-Cre* mice.

To induce hepatocyte apoptosis, 4-wk-old mice were injected intraperitoneally with 10 µg of purified NA/LE hamster antimouse CD95 (Clone Jo-2, BD Biosciences 554254). Liver samples were analyzed 3 h after injection. To induce acute liver injury, a single intraperitoneal injection of 20% carbon tetrachloride (CCl_4_; Sigma 289116) dissolved in corn oil at a dose of 1 mL/kg was performed in 3-mo-old animals. Liver samples were analyzed 2, 4, and 6 d after CCl_4_ injection. Animal protocols were approved by the Institutional Animal Care and Use Committee of the University of Texas Southwestern Medical Center.

### RNA-seq analysis

Total RNAs were extracted from livers of three 1-mo-old control, *Vgll4* mutant, *Yap* mutant, and *Yap;Vgll4* double-mutant mice using the TRIzol reagent (Ambion 15596018) and cleaned up with a Turbo DNA-free kit (Ambion AM1907). Samples were run on an Agilent 2100 Bioanalyzer for quality control. One microgram of RNA from each sample was prepared with a TruSeq stranded total RNA LT sample preparation kit from Illumina. cDNAs were synthesized after removal of ribosomal RNAs, ligated with adapters, and amplified. Samples were run on an Illumina NextSeq 500 system at the University of Texas Southwestern Next-Generation Sequencing Core. Sequencing reads were mapped to the mm10 mouse genome, and differential gene expression analysis was carried out with the edgeR R package (v.3.36.0). Genes with a change in expression of at least twofold (*P*-value < 0.05) were visualized using heat maps. GO enrichment analysis was performed at the ShinyGO 0.76.1 website (http://bioinformatics.sdstate.edu/go) with FDR < 0.05.

### Serum alanine aminotransferase (ALT) and direct bilirubin measurement

Approximately 200 μL of whole blood from the submandibular vein was collected into a Microvette tube coated with heparin (Sarstedt 16.443.100). Plasma was collected after centrifugation at 1000*g* for 10 min using a refrigerated centrifuge. The levels of ALT and direct bilirubin in plasma were analyzed by Vitros MicroSlide Technology at the University of Texas Southwestern Medical Center Metabolic Phenotyping Core.

### Western blotting

Livers were lysed, and the extracted proteins were analyzed. The primary antibodies used for Western blot were rabbit anti-PARP (1:1000; BD Biosciences 556362), anti-cleaved Caspase-3 (1:1000; Cell Signaling 9661), anti-Actin (1:20,000; Millipore MAB1501R), anti-YAP (1:1000; Cell Signaling 4912), anti-p-YAP (1:1000; Cell Signaling 4911), and anti-GAPDH (1:1000; Cell Signaling). YAP, p-YAP, and GAPDH signals were quantified by ImageJ.

### Mouse histological analysis and immunohistochemistry

Mouse livers were collected, fixed overnight in 4% paraformaldehyde in 1× PBS, embedded in paraffin, and sectioned at 5 μm. Sections were stained with hematoxylin and eosin for histological analysis. Sirius red (Abcam ab150681) staining and immunohistochemistry were performed according to the manufacturers’ protocols. Primary antibodies used for immunohistochemistry were rabbit anti-Ki67 (1:500; Novocastra NCL-Ki67p), anti-cleaved Caspase-3 (1:100; Cell Signaling 9661), anti-PanCK (1:200; DAKO Z0622), anti-Sox9 (1:200; Millipore AB5535), and anti-α-SMA (1:200; Sigma C6198). For Ki67, the signals were developed using the ABC kit purchased from Vector Laboratories according to the manufacturer's suggestions. Cy3-conjugated goat antirabbit secondary antibodies (Molecular Probes) were used for immunofluorescence.

### Quantitative real-time PCR

RNA from livers was extracted using the TRIzol reagent (Ambion 15596018). RNA was reverse-transcribed using the iScripTM cDNA synthesis kit (Bio-Rad 1708891). Quantitative real-time PCR was performed using the iQ SYBR Green supermix (Bio-Rad 1708882) on a CFX96 real-time system (Bio-Rad). Primers for real-time PCR are listed in Supplemental Table S3.

## Supplementary Material

Supplemental Material
