# Supplementary material for: YAP–VGLL4 antagonism defines the major physiological function of the Hippo signaling effector YAP
Source: Genes Dev. 2022 Nov-Dec;36(21-24):1119–28. doi: 10.1101/gad.350127.122 (PMC9851404; doi:10.1101/gad.350127.122)
Supplement: Supplemental Material [file supp_36_21-24_1119__DC1.html]

YAP–VGLL4 antagonism defines the major physiological function of the Hippo signaling effector YAP — YAP–VGLL4 antagonism defines the major physiological function of the Hippo signaling effector YAP — Supplemental Material 

# YAP–VGLL4 antagonism defines the major physiological function of the Hippo signaling effector YAP

## Supplemental Material

- Supplemental\_Data.pdf
- Supplemental\_Table\_1.xlsx
- Supplemental\_Table\_2.xlsx
