# Supplementary material for: YAP–VGLL4 antagonism defines the major physiological function of the Hippo signaling effector YAP
Source: Genes Dev. 2022 Nov-Dec;36(21-24):1119–28. doi: 10.1101/gad.350127.122 (PMC9851404; doi:10.1101/gad.350127.122)
Supplement: Supplemental Material [file supp_gad.350127.122_Supplemental_Data.pdf]

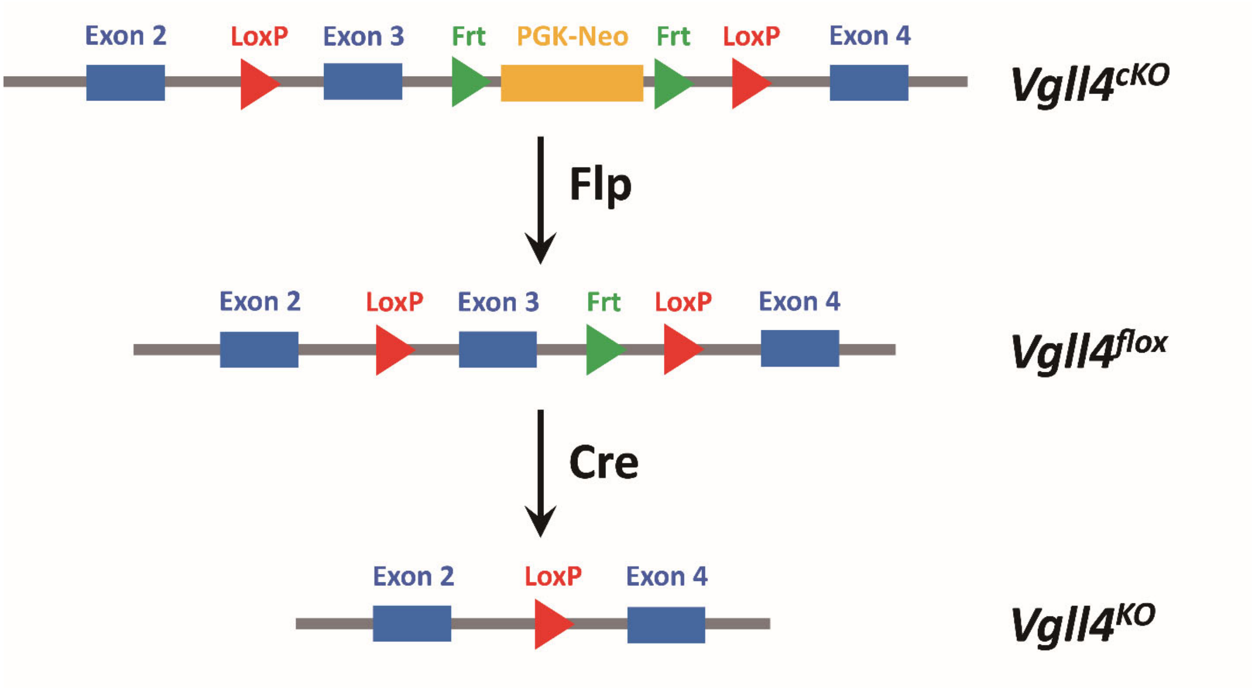

Supplemental Figure 1. Generation of *Vgll4* conditional knockout mice.

Targeting strategy. Exons 3 of the *Vgll4* gene is removed by Cre-mediated recombination.

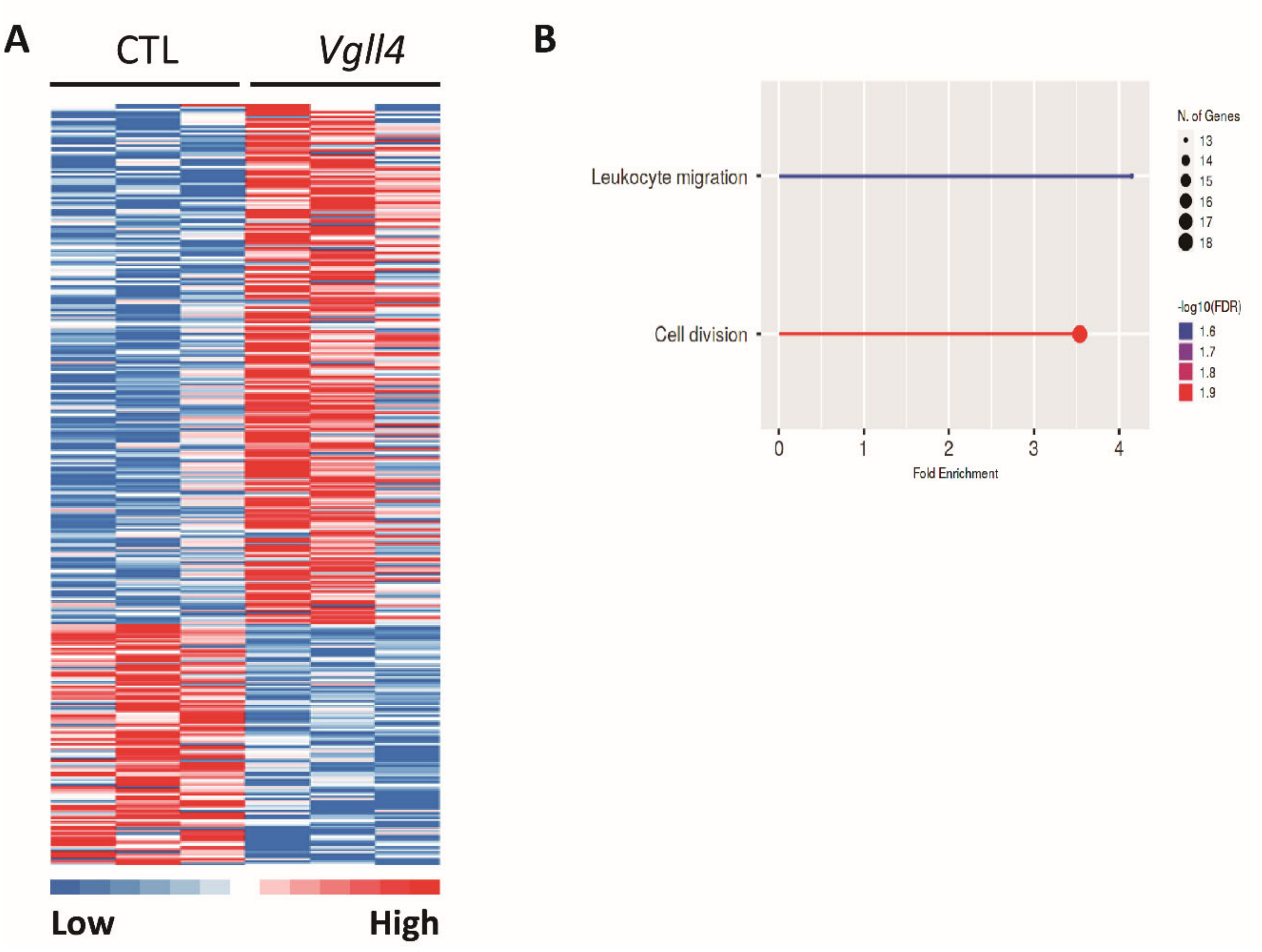

Supplemental Figure 2. Gene expression profiling of 1-month-old control and *Vgll4* mutant livers by RNA-seq.

A. Heat map analysis of the 218 up-regulated genes and 101 down-regulated genes in *Vgll4* mutant livers compared to the control. Livers from three independent mice of each genotype were analyzed.

B. The enriched pathways as detected by GO enrichment analysis of the 218 up-regulated genes.

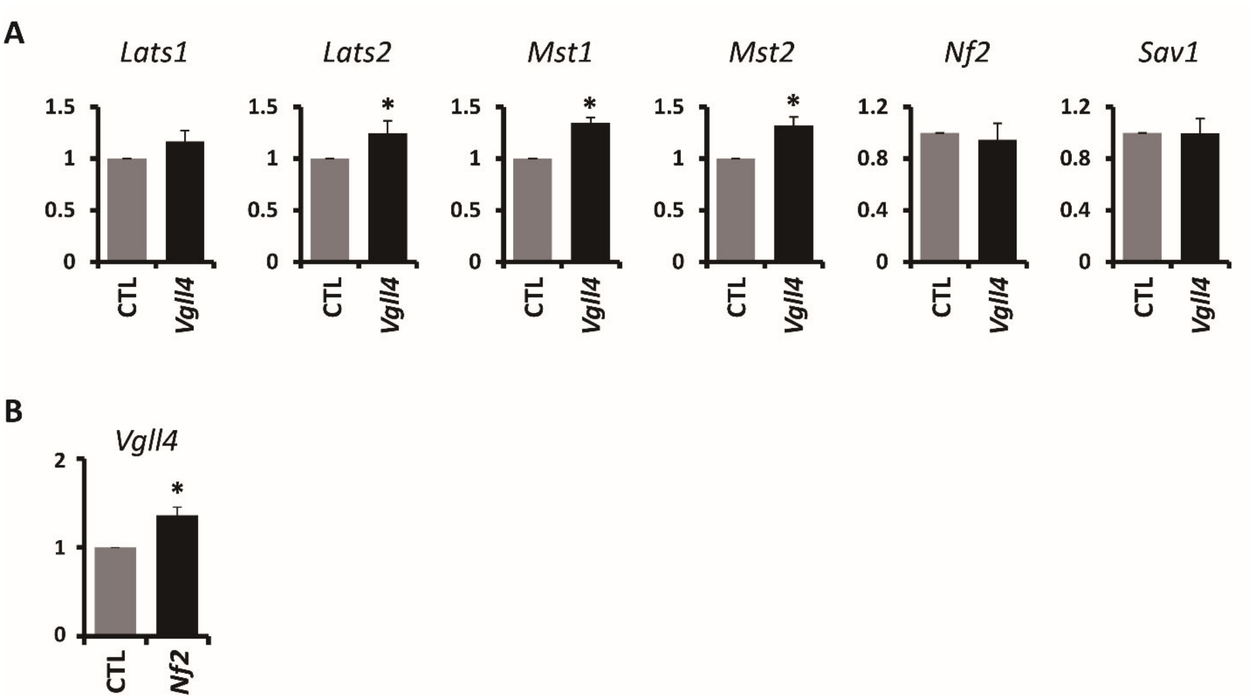

Supplemental Figure 3. Compensatory regulation of Hippo signaling components and Vgll4 in *Vgll4* and *Nf2* mutant livers.

A. Up-regulation of upstream regulators of YAP, including *Lats2*, *Mst1* and *Mst2*, in 1-month-old *Vgll4* mutant livers. Data are mean ± SD. n = 3. (*) P < 0.05, t-test.

B. Up-regulation of *Vgll4* in 1-month-old *Nf2* mutant livers. Data are mean ± SD. n = 3. (*) P < 0.05, t-test.

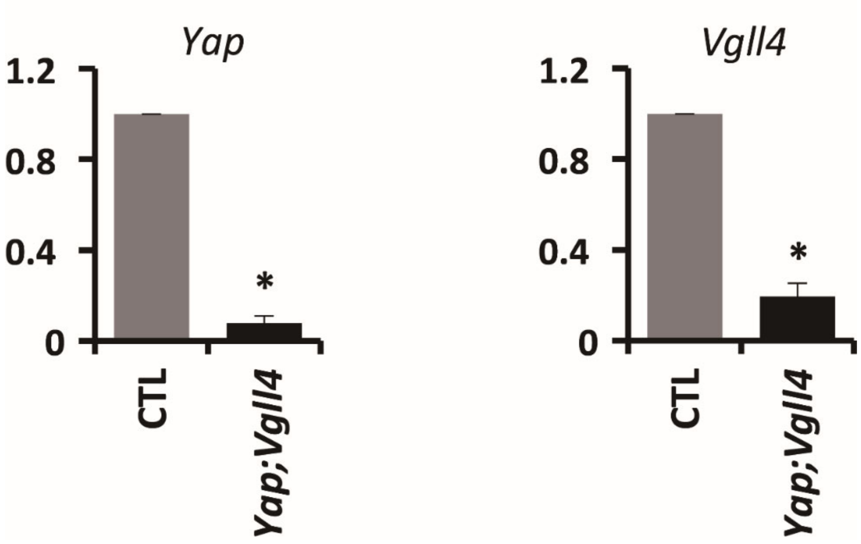

Supplemental Figure 4. Confirmation of deleting *Yap* and *Vgll4* in *Yap;Vgll4* mutant livers. Down-regulation of *Yap* and *Vgll4* in *Yap;Vgll4* mutant livers. Data are mean ± SD. n = 3. (*) $P < 0.01$, t-test.

Supplementary Table 1. Fold change of genes differentially expressed in *Vgll4* mutant compared to control livers.

See separate Excel document uploaded as a supplemental file.

Supplementary Table 2. Fold change of genes differentially expressed in *Yap* mutant compared to control livers, and their rescue in *Yap*;*Vgll4* double mutant livers.

See separate Excel document uploaded as a supplemental file.

Supplementary Table 3. Primer sequences for real-time PCR.

Primer	Sequence
c-Myc forward	CCTTTGGGCGTTGGAAACC
c-Myc reverse	CGTCGCAGATGAAATAGGG
Ctgf forward	AGTGTGCACTGCCAAAGATG
Ctgf reverse	CCAGGCAAGTGCATTGGTAT
Cyr61 forward	GCTCAGTCAGAAGGCAGACC
Cyr61 reverse	GTTCTTGGGGACACAGAGGA
Amotl2 forward	AGGGACAATGAGCGATTGCAG
Amotl2 reverse	CCTCACGCTTGGAAGAGGT
Ankrd1 forward	TGCGATGAGTATAAACGGACG
Ankrd1 reverse	GTGGATTCAAGCATATCTCGGAA
Birc5 forward	GACAACCCGATAGAGGAG
Birc5 reverse	TCAGGTCCAAGTTATCTCAG
Lats1 forward	TGGTGACTCTGGGGATAAAGAA
Lats1 reverse	GGGAGTAACTCTGAATCCGAGAC
Lats2 forward	GGACCCCAGGAATGAGCAG
Lats2 reverse	CCCTCGTAGTTTGCACCACC
Mst1 forward	CAGTAATAGGGACCCCGTTTTG
Mst1 reverse	GGGCTTTCCTTCAGCCATTTC
Mst2 forward	CCGGCGTCCAAGAGTAAGC
Mst2 reverse	GCAACCACTTGACCAGATTCC
Nf2 forward	CTAGTTCAAGAGATCACGCAACA
Nf2 reverse	GGCAGTAGACCTTTTCATCCAAA
Sav1 forward	CTGTCCCGCAAGAAAACCAAA
Sav1 reverse	AATGAAGGCATGAGATTCCGC
Yap forward	TACATAAACCATAAGAACAAGACCACA
Yap reverse	GCTTCACTGGAGCACTCTGA
Vgll4 forward	TGTGAAAACGACCACGTCTCC
Vgll4 reverse	GCAGTCTCCGTTGACAGTCTTAT
Gapdh forward	CCCAATGTGTCCGTCGTGGAT
Gapdh reverse	TGTAGCCCAAGATGCCCTTCAG
